# CowPI: A Rumen Microbiome Focussed Version of the PICRUSt Functional Inference Software

**DOI:** 10.3389/fmicb.2018.01095

**Published:** 2018-05-25

**Authors:** Toby J. Wilkinson, Sharon A. Huws, Joan E. Edwards, Alison H. Kingston-Smith, Karen Siu-Ting, Martin Hughes, Francesco Rubino, Maximillian Friedersdorff, Christopher J. Creevey

**Affiliations:** ^1^The Roslin Institute and R(D)SVS, University of Edinburgh, Edinburgh, United Kingdom; ^2^Institute of Biological, Environmental and Rural Sciences, Aberystwyth University, Aberystwyth, United Kingdom; ^3^Medical Biology Centre, School of Biological Sciences, Queen’s University Belfast, Belfast, United Kingdom; ^4^Animal Nutrition Group, Wageningen University and Research, Wageningen, Netherlands; ^5^Animal and Bioscience Research Department, Teagasc, Grange, Ireland

**Keywords:** PICRUSt, CowPI, rumen, function, 16S amplicon

## Abstract

Metataxonomic 16S rDNA based studies are a commonplace and useful tool in the research of the microbiome, but they do not provide the full investigative power of metagenomics and metatranscriptomics for revealing the functional potential of microbial communities. However, the use of metagenomic and metatranscriptomic technologies is hindered by high costs and skills barrier necessary to generate and interpret the data. To address this, a tool for Phylogenetic Investigation of Communities by Reconstruction of Unobserved States (PICRUSt) was developed for inferring the functional potential of an observed microbiome profile, based on 16S data. This allows functional inferences to be made from metataxonomic 16S rDNA studies with little extra work or cost, but its accuracy relies on the availability of completely sequenced genomes of representative organisms from the community being investigated. The rumen microbiome is an example of a community traditionally underrepresented in genome and sequence databases, but recent efforts by projects such as the Global Rumen Census and Hungate 1000 have resulted in a wide sampling of 16S rDNA profiles and almost 500 fully sequenced microbial genomes from this environment. Using this information, we have developed “CowPI,” a focused version of the PICRUSt tool provided for use by the wider scientific community in the study of the rumen microbiome. We evaluated the accuracy of CowPI and PICRUSt using two 16S datasets from the rumen microbiome: one generated from rDNA and the other from rRNA where corresponding metagenomic and metatranscriptomic data was also available. We show that the functional profiles predicted by CowPI better match estimates for both the meta-genomic and transcriptomic datasets than PICRUSt, and capture the higher degree of genetic variation and larger pangenomes of rumen organisms. Nonetheless, whilst being closer in terms of predictive power for the rumen microbiome, there were differences when compared to both the metagenomic and metatranscriptome data and so we recommend, where possible, functional inferences from 16S data should not replace metagenomic and metatranscriptomic approaches. The tool can be accessed at http://www.cowpi.org and is provided to the wider scientific community for use in the study of the rumen microbiome.

## Introduction

Ruminant livestock represent an important part of human nutrition as a major source of our meat and milk (Webb et al., 2011). The functionality of the rumen microbial population allows the conversion of plant material of relatively low nutritional value into readily absorbed vital compounds for the animal. Furthermore, the efficiency of the different fermentation processes employed by the rumen microbial community dictates the quality and quantity of production within each animal (Mackie, 2002; Edwards et al., 2008; Kingston-Smith et al., 2010). As the demand for ruminant food products increases, so too does the need to maximize this production in relation to cost and animal welfare, and with as little negative impact on the environment as possible (FAOSTAT, 2009).

Next generation sequencing (NGS) has proved to be an invaluable tool in progressing the study of the rumen microbiome. The majority of studies focus on metataxonomics using amplicon libraries of marker genes, such as the 16S rDNA gene, and assessing change in the community structure in relation to animal productivity in different ruminant species and breeds (Myer et al., 2015; De Mulder et al., 2017), and in response to dietary intervention (Yáñez-Ruiz et al., 2015; Belanche et al., 2016). Metataxonomic and metagenomic approaches have also been used to investigate more complex plant-microbe interactions and colonization of forage (Piao et al., 2014; Huws et al., 2016; Mayorga et al., 2016). Despite advancement of sequencing technology and analysis methodology to study the full metagenomic and metatranscriptomic profiles of ruminal microbiomes (Li et al., 2012; Kamke et al., 2016), this type of study remains computationally complex and a financially demanding undertaking, whereas metataxonomic studies are comparatively cheaper (Jovel et al., 2016).

Using the 16S rDNA metataxonomic approach (Marchesi and Ravel, 2015) it is possible to correlate changes in the abundance of certain bacteria with functional changes, but these estimates are often crude due to the functional variation that can be found within genera (Piao et al., 2014; Belanche et al., 2016; Huws et al., 2016; McInerney et al., 2017; Rubino et al., 2017). Indeed, recent studies investigating the extent of diversity in prokaryote pangenomes have revealed a strong correlation between the ratio of core to accessory genome and the “lifestyle” of the prokaryote species and suggest that a larger pangenome and higher proportion of accessory genes allows prokaryotes to fill more environmental niches (McInerney et al., 2017). Within the rumen microbiome the two most abundant bacterial genera, *Prevotella* and *Clostridium* have also been shown to contain significant differences in functional isoforms in at least 153 genes important to their niche (Rubino et al., 2017).

Given the need to understand bacterial function and the cost restrictions of metagenomic and metatranscriptomic approaches, the Phylogenetic Investigation of Communities by Reconstruction of Unobserved States (PICRUSt) software was developed to take the predictive inference a step further. This is done by taking well-known fully characterized bacterial genomes and uses their phylogenetic relationships to predict the functional genome of other bacteria within the constructed phylogeny based on 16S rDNA data (Langille et al., 2013). Having been validated with data from the Human Microbiome Project, the tool has been applied to the study of environmental microbiomes (Hartman et al., 2017; Ren et al., 2017), gut microbiota (Reed et al., 2017; Wilkinson et al., 2017), and, indeed, within the rumen (Meale et al., 2016; Popova et al., 2017). However, the original PICRUSt implementation is based on a wide selection of microbial genomes primarily from the human microbiome (Langille et al., 2013) which may reduce the accuracy of functional predictions when applied to data from other microbiomes.

The rumen microbiome is a case in point; organisms from this environment are traditionally underrepresented in genome and rRNA sequence databases. However, recent international efforts by projects such as the Global Rumen Census (GRC) (which generated a global 16S rRNA-based census of rumen microbial constituents, Henderson et al., 2015) and the Hungate 1000 (which has cultured and sequenced over 400 rumen microbial genomes, Seshadri et al., 2018) presents an opportunity to develop a version of PICRUSt which is solely based on data from the rumen microbiome (Creevey et al., 2014). Although the resulting genomes are fewer in number than used in the original PICRUSt implementation, they represent a more focused starting dataset from which to construct ancestral state trait predictions and infer potential microbiome function from rumen metataxonomic studies.

Here we describe a “rumen-specific” version of the underlying PICRUSt pre-calculated files generated with data from the GRC and Hungate 1000 projects, using the ancestral state reconstruction methods for genome prediction as provided with the PICRUSt software. We assess the accuracy of this bespoke implementation by comparing the results obtained from 16S rDNA and rRNA rumen bacterial metataxonomic studies, where corresponding metagenomic and metatranscriptomic data was also available. The precalculated files are available upon request from the authors and the tool, is offered for use by the wider community in the study of the rumen microbiome at http://www.cowpi.org.

## Materials and Methods

### Generating Pre-calculated Files

PICRUSt provides users with the scripts necessary to create trait predictions for their own annotated genomes. These scripts require the user to generate three files fundamental to the use of this workflow: (i) a marker gene copy number table containing 16S copy numbers for each bacterial genome; (ii) functional gene copy number table using KEGG (Kanehisa and Goto, 2000) Ortholog counts for each bacterial genome; (iii) a reference tree which is a phylogenetic reconstruction using 16S sequences and contains tips representing sequenced and non-sequenced genomes.

Assembled reads (contigs/scaffolds) for 497 rumen microbial genomes (see Supplementary Table S4 for more details) were downloaded from JGI or NCBI (accessed January 2017). PROKKA (v1.12, Seemann, 2014) was used to annotate each genome, and only genes with a Uniprot ID were considered. The Bioconductor package KEGGREST (v1.17.1, Tenenbaum, 2017) was used in R to extract KEGG ortholog (KO) IDs, using the unique Uniprot IDs, and create a frequency count of KO IDs present in each Hungate1000 genome. HMMER identification of 16S genes in PROKKA were used to create a count table of 16S copy numbers for each genome (see Supplementary Table S4). KEGG BRITE functional hierarchical data was also extracted for each KO and added as a “metadata” value to the end of the count table. Two KO count tables were produced, one that contained only KOs present in the count table supplied with the PICRUSt package and a further table with duplicated columns for KOs that have multiple entries in the KEGG BRITE database, something that is not accounted for in the original PICRUSt data. A phylogenetic tree was constructed from 696,451 16S rDNA sequences collected as part GRC, as well as sequences from the 497 Hungate1000 genomes. These files were then used as input to the Genome Prediction workflow using the format_tree_and_trait_table.py, ancestral_state_reconstruction.py and predict_traits.py scripts as supplied with PICRUSt, all with default parameters.

### The CowPI Workflow

To utilize PICRUSt users must provide an OTU table with IDs present in the GreenGenes database (DeSantis et al., 2006) usually produced by closed OTU picking in QIIME (Caporaso et al., 2010). As CowPI uses a custom underlying database of 16S sequences from rumen microorganisms (from the GRC), the provided table must utilize IDs present from this set of 16S rDNA data. Here we provide a custom step to allow this classification which also provides a solution for CowPI users who have not used QIIME for OTU picking and clustering. This involves the use of a fasta formatted file of consensus/representative sequences for each OTU from the combined GRC/Hungate 1000 16S sequences along with table of OTU abundances from the dataset to be analyzed. These are used as input to mothur (Schloss et al., 2009) or usearch (Edgar, 2010), to classify the unknown 16s sequences against the 696,451 GRC sequences and a script in R is then used to sum the values for OTUs with common classifications. The resultant file is then converted to biom format ready for the standard PICRUSt steps for metagenome prediction using the KO frequencies calculated for the hungate 1000 genomes. The tools to carry out these steps are all provided in the galaxy implementation of CowPI.

### Validation Study

We assessed the accuracy of both CowPI and PICRUSt in predicting the functional potential of a rumen microbiome using the 16S rDNA data from the study by Hess et al. (2011). Critically, this study also carried out metagenomic sequencing allowing us to compare the predictions of both tools to the real functional profile. We obtained a set of predicted protein sequences from the Hess et al. (2011) rumen metagenomic dataset by contacting the lead author. This dataset had 2,547,270 predicted proteins, which were then annotated with PROKKA, and all resulting Uniprot IDs were mapped to predicted Kegg orthologs (KO) using the uniprot mapping tool^1^. allowing the construction of a KO frequency table for the metagenomic data. Complete data can also be accessed through the Web site of the DOE Joint Genome Institute^2^. Metataxonomic data from the same samples was also obtained from the lead author consisting of a set of OTU counts and representative sequences for each OTU cluster. These were used as input for both the PICRUSt and CowPI workflows. To more directly compare the two predicted metagenomes (CowPI and PICRUSt) to the sequenced metagenome, counts for KOs only present in all three datasets were compared. Using the relative abundance of each KO, Pearson correlations were calculated between each predicted metagenome and the observed, sequenced metagenome, using R. Plots representing the correlation were constructed using ggplot2 in R (Wickham, 2009).

### Comparative Studies

To understand the performance of CowPI and PICRUSt for generating functional predictions for transcriptome-based data, the RNA based metataxonomic data from Huws et al. (2016) (NCBI bioproject ID PRJNA274256) was analyzed using both approaches and compared to the metatranscriptomic data that was generated for the same samples. This experiment examined the colonization profile of microbes on grass following igestion by the cow, using three cows as replicates and examining five time points following ingestion (1, 2, 4, 6, and 8 h). We present here a complete analysis of this dataset comparing the results from CowPI, PICRUSt and a metatranscriptome to identify differences in predicted functional capacity of the microbiome over time. This also allowed demonstration of the types of analyses (using multiple replicated samples and time-points) that are possible to carry out using the CowPI workflow.

An explanation of the experimental design and metataxonomic workflow are described in Huws et al. (2016). In brief, using the nylon bag method, fresh perennial ryegrass was incubated in the rumen of three cannulated, non-lactating Holstein x Friesian cows. Two bags were removed at 1, 2, 4, 6 and 8 h post incubation, and residual forage was washed and stored at -80°C. In order to obtain metatranscriptome data, rumen samples from the experiment were frozen and ground to a fine powder under liquid nitrogen before RNA was extracted using a hot phenol method (Ougham and Davies, 1990). Essentially aquaphenol (10 mL) was added to the ground sample prior to incubation at 65°C for 1 h. Tubes were inverted before chloroform was added (5 mL). Tubes were centrifuged (5,000 ×*g*, 30 min, 20°C) before upper phase was removed then the procedure was repeated by addition of more chloroform (5 mL) and centrifugation as described. Lithium chloride (2 M final concentration) was then added, to remove any contaminating DNA, and samples stored overnight at 4°C. Samples were subsequently centrifuged (13,000 ×*g*, 30 min, 4°C) and supernatant discarded, then the procedure was repeated from addition of lithium chloride to ensure all DNA was removed. Once the supernatant was discarded the pellet was resuspended in ice cold 80% ethanol and centrifuged (13,000 ×*g*, 15 min, 4°C), this was repeated twice before the pellet was air dried and resuspended in molecular grade water. Absence of DNA in all sample RNA extracts was checked using PCR as described in Huws et al. (2016), using non-barcoded primers and subsequent agarose gel electrophoresis. Quality and quantity of retrieved RNA was checked using the Experion automated electrophoresis system and RNA StdSens chips (Bio-rad, Hemel Hempstead, United Kingdom). Bacterial mRNA was enriched in all samples by firstly removing the polyA fraction (MicroPoly(A)Purist, Ambion), according to the manufacturer’s protocol. Then eukaryotic 18S rRNA was removed using both RiboMinus Plant Kit and the eukaryote kit (Invitrogen, Paisley, United Kingdom), according to manufacturer’s protocols. Finally, 16S rRNA [Ribo-Zero rRNA removal kit (bacteria), Epicentre] was removed according to manufacturer’s protocols. Resultant enriched mRNA was prepared for sequencing using TruSeq stranded mRNA library prep kit (Illumina, California, United States) following manufacturer’s guidelines. Subsequently, library sequencing was completed using the Illumina HiSeq 2500 (Illumina, California, United States) (100 bp paired end sequencing), data is deposited in the NCBI repository under bioproject ID PRJNA419191.

Before assembly, the mRNA reads were filtered for plant material and known references such as those from the Hungate 1000, using bowtie2 (Langmead and Salzberg, 2012). The remaining reads were trimmed according to their quality using FastQC (Andrews, 2010) and subsequently normalized by median using the khmer package (Crusoe et al., 2015). Afterwards, the reads were partitioned using the k-mer and finally assembled using velvet (Zerbino and Birney, 2008). Assembled contigs were subjected to the same annotation procedure as the Hungate 1000 genomes using PROKKA, extracting Uniprot and KO IDs to produce a KO/sample count table. Amplicon reads were clustered at 97% similarity using CD-HIT-OTU to produce a typical input OTU table and fasta formatted file of sequences representing each OTU. These were used as input for both the PICRUSt and CowPI workflows. The profiles of functional pathways, blocked by time point were subjected to multiple group ANOVA, Tukey–Kramer *post hoc* analysis and corrected for multiple testing with the Benjamini–Hochberg method using the software package STAMP: statistical analysis of taxonomic and functional profiles (Parks et al., 2014). Principal components of Euclidean distances between pathway profiles of samples were also analyzed using STAMP. To more directly compare the two predicted metatranscriptomes (CowPI and PICRUSt) to the sequenced metatranscriptome, counts for KOs only present in all three datasets were combined. Using the Bioconductor package DESeq2 in R (Love et al., 2014) with the samples grouped by time and metatranscriptome set (CowPI, PICRUSt or sequenced metatranscriptome), counts were regularized log transformed, principal components calculated, and finally plotted using ggplot2 in R. As has been previously shown there is a major change in the bacterial community attached to perennial rye grass in the rumen between 2 and 4 h of incubation. It is hypothesized that the change in community follows the degradation of hemicellulose by the primary colonizers, moving to cellulose metabolism by the secondary colonizers and represents a significant change in metabolic function of the community (Huws et al., 2016). To provide greater resolution in the investigation of difference in functional potential, using DESeq2, KOs that were significantly (corrected *P* (FDR) < 0.1) differentially abundant when contrasting the 2 and 4 h time point samples, were subjected to over-representation analysis, and dotplots produced, using the Bioconductor packages clusterProfiler (Yu et al., 2012) and DOSE (Yu et al., 2015) in R. *P* values were again corrected for multiple testing using the Benjamini–Hochberg method.

## Results

### Validation Study

**FIGURE 1:**
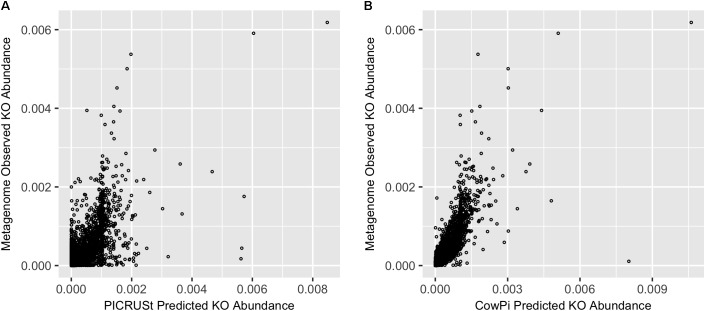
Prediction accuracy on the Hess et al. (2011) dataset. **(A)** Metagenome compared with PICRUSt and **(B)** Metagenome compared with CowPI. Points represent the relative abundance of KOs in the observed (*y*-axis) and predicted (*x*-axis) dataset.

Comparison of PICRUSt and CowPI predicted metagenomes with the sequenced annotated observed metagenome resulted in 5901 KOs that were predicted to be present in all three datasets (CowPI, PICRUSt and the metagenome). PICRUSt predicted KO abundance was moderately correlated with metagenome observed KO abundance (**Figure 1**) with a significant (*p* < 0.05) Pearson correlation of *R*^2^ = 0.420, CowPI predicted KO abundance was also significant (*p* < 0.05) but with stronger correlation with the observed metagenome KO abundance *R*^2^ = 0.647 (**Figure 1**).

### Pathway Level

ANOVA of pathways present in the metatranscriptome showed that 28 were significantly different (*P* < 0.05) between time points. Analysis of the 16S rDNA sequences using the standard PICRUSt pre-calculated files, the number of significant pathways increases to 87. Analysis of the same data using the CowPI workflow results in 40 significantly different pathways (**Table 1**). Of the significant pathways, those involved in metabolism represent 64.29%, 56.32%, and 61.54% in the metatranscriptome, PICRUSt, and CowPI predictions, respectively. Pathways involved in cellular processes and signaling make up 14.29%, 3.45%, and 2.56% of significant pathways. The metatranscriptome analysis didn’t identify any pathways involved in the processing of genetic information, whereas PICRUSt predicted 12 such pathways to be significant (13.79%) and CowPI predicted 4 (10.26%) (**Table 1**). Of these significant pathways, the number related to metabolism is 18 in the metatranscriptome, 24 in CowPI, and 49 in PICRUSt, (Supplementary Tables S1–S3).

**Table 1:** Significant Pathways in each dataset, numbers in parentheses represent percentage of significant pathways within that dataset.

Pathway	Transcriptome	PICRUSt	CowPI
KOs	1521	6909	4585
Sig. pathways	28	87	40
Genetic information processing	0 (0)	12 (13.79)	4 (10.26)
Metabolism	18 (64.29)	49 (56.32)	24 (61.54)
Cellular processes and signaling	4 (14.29)	3 (3.45)	1 (2.56)
Human diseases	0 (0)	7 (8.05)	7 (17.95)
Organismal systems	1 (3.57)	7 (8.05)	2 (5.13)
Cellular processes	3 (10.71)	5 (5.75)	0 (0)
Environmental information processing	1 (3.57)	2 (2.3)	1 (2.56)
Poorly characterized	1 (3.57)	2 (2.3)	0 (0)

**FIGURE 2:**
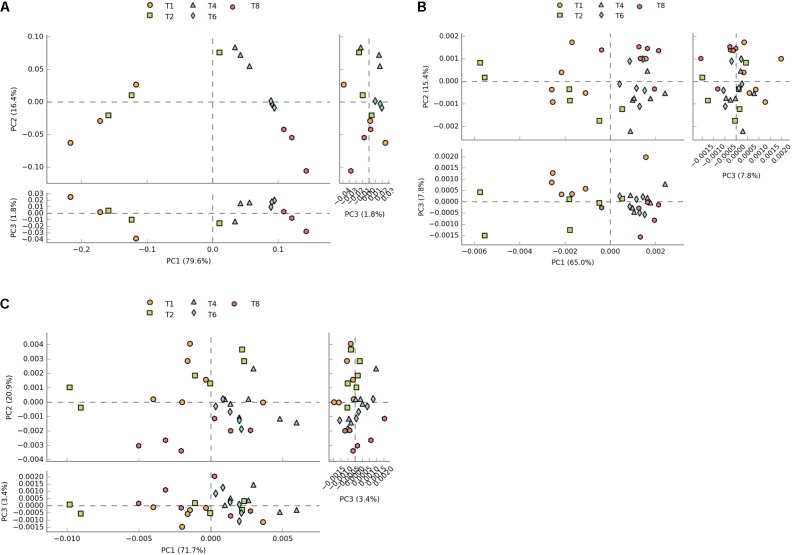
Plots of principal components of distances between sample pathway profiles. **(A)** PC1 accounts for 79.6% and PC2 16.4% of the variation between samples in the transcriptome dataset. **(B)** PC1 accounts for 65% and PC2 for 15% of variation in the PICRUSt predicted pathways. **(C)** For the CowPI predicted pathways, PC1 represents 71.7% of the variation and PC2, 20%.

Due to KOs being potentially involved in multiple pathways, and not necessarily identified by the downstream analysis of PICRUSt data, pathways related to KEGG modules with seemingly no relevance to microbiome analysis such as Human Diseases and Organismal Systems were not removed from the datasets to allow a more direct comparison of predicted counts. Principal component analysis of profiles of functional pathways of the metatranscriptome data set shows clear separation between 1 and 2 h time points and later time points (**Figure 2A**). This separation is less evident in the PICRUSt (**Figure 2B**) and CowPI (**Figure 2C**) datasets, although this separation seems to be more accounted for by PC1 when using CowPI predicted pathways (**Figure 2**).

### KO Level

**FIGURE 3:**
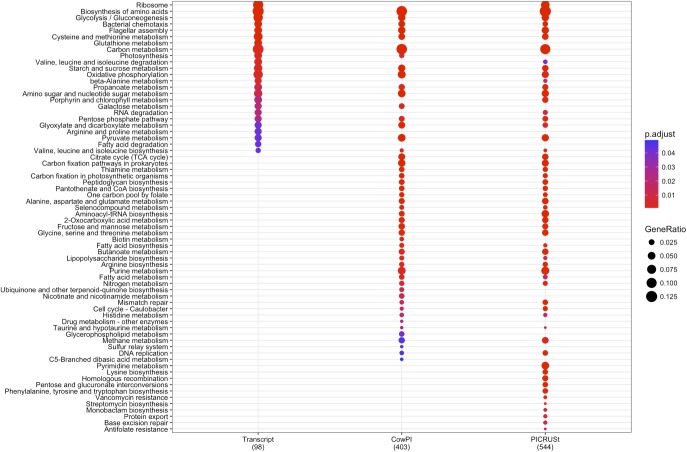
Significantly over-represented KEGG functional hierarchies by differentially abundant KEGG orthologs (KOs) between 2 and 4 h samples for each dataset. Circle size represents KO counts within that pathway in proportion to the total number of differentially abundant KOs, while red coloration represents lower *P* value and therefore higher significance. Numbers in parentheses are the total number of KOs contributing to the analysis.

**FIGURE 4:**
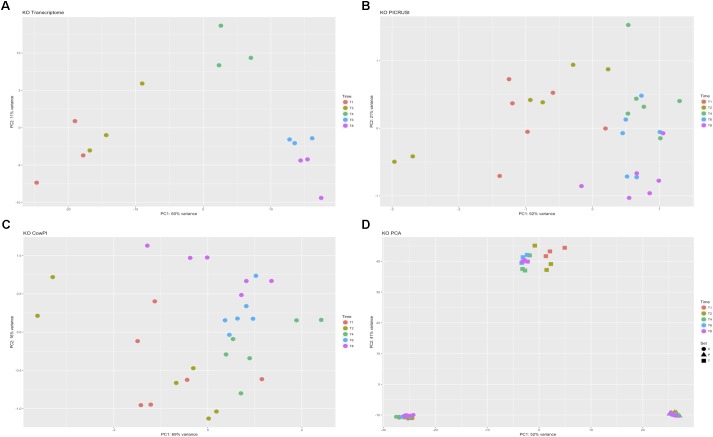
Principal component analyses of KO counts present only in all three datasets. **(A)** Transcriptome dataset, **(B)** PICRUSt predicted KOs, **(C)** CowPI predicted KOs, **(D)** analysis including all samples blocked by time point and sample set.

DESeq2 highlighted 98 KOs that are significantly differentially abundant between 2 and 4 h samples in the metatranscriptome data set whereas CowPI analysis produced 403 and PICRUSt 544. Over-representation analysis of the genes associated with these differentially abundant KOs indicated that there were 24, 49, and 56 significantly represented pathways in the metatranscriptome, CowPI, and PICRUSt datasets, respectively (**Figure 3**). PC1 accounted for the most variation between samples in all three data sets. PC2 accounted for less variation in the metatranscriptome and CowPI datasets but more in the PICRUSt. Separation and grouping is largely the same as in the pathway level analyses. When KO counts from all samples across all three data sets are analyzed together, separation by analysis type is clearly seen (**Figure 4**).

## Discussion

Community profiling techniques have always represented an invaluable tool in the study of the rumen microbiome and recent advancements in NGS have allowed a greater number of researchers across the globe, access to this methodology. As a result, however, traditional culture-based approaches have fallen out of favor. While we may now know more about the range of taxa present and the community dynamics in relation to treatments and environmental variables, metataxonomics provides little information in relation to rumen functionality. Global projects, such as the GRC and the Hungate 1000 aim to close this gap in knowledge, providing a more comprehensive outline of the true species variation and making a significant step forward in available genomic and functional information regarding the complex rumen microbiome.

Early analysis of the data produced by the Hungate1000 by Creevey et al. (2014), highlighted surprisingly low levels of genomic similarity between some members of the rumen microbiome and closely related organisms available in public databases. Authors found that many taxa in the major rumen bacterial phylum *Bacteroidetes* had very low representation in the databases, and showed only weak homology to families such as *Rikenellaceae* and *Porphyromonadaceae*. Furthermore, the highly abundant genus *Prevotella* is made up of two groups: (i) species that cluster with other environmentally isolated species of *Prevotella* and (ii) a group of species generally only found in the rumen environment that show very weak homology to well-known cultured isolates. Given the lack of function-based inference from metataxonomic data, and the costs and labor associated with obtaining metagenomic and metatranscriptomic datasets, the PICRUSt software was developed to allow better functional inferences to be made from 16S rDNA data. Nonetheless, PICRUSt was developed for human microbiome studies and although used in rumen microbiome studies, the quality of the functional inferences made is unknown.

In this study, we compared the performance of PICRUSt to a bespoke PICRUSt implementation, adapted for rumen microbiome studies (CowPI) using the commonly referenced rumen dataset published by Hess et al. (2011) that had both 16S rDNA data and full metagenome data available. Results from this comparison deomstrate that CowPI more accurately predicts potential functionality of the metagenome than PICRUSt. Previously PICRUSt has been shown to correlate well with human and environmental datasets (Pearson *R*^2^ = 0.787, Langille et al., 2013; Supplementary Data), however, when we applied this to the rumen dataset from Hess et al. (2011), PICRUSt performed poorly (Pearson *R*^2^ = 0.420). In contrast using the data from the GRC and Hungate 1000 projects, the CowPI predicted KO abundance of the microbiome displayed a much greater correlation with metagenome observed KO abundance (Pearson *R*^2^ = 0.647).

To better assess the functional predictions in an experimental context, we also analyzed 16S rDNA metataxonomic data and compared the predictions of CowPI and PICRUSt to the functional predictions based on metatranscriptome data from the same study. Our results show that the functional predictions of metabolic activity in the rumen microbiome is improved when using CowPI compared to PICRUSt. While both inference methods over-predicted the number of pathways with significant difference over time compared to the metatranscriptome dataset CowPI was less affected by this bias than PICRUSt and produced results closer to that found from the metatranscriptome data. Similarly, within the Metabolism KEGG module, CowPI showed more similarity to the metatranscriptome in numbers of significant pathways related to carbohydrate, lipid, amino acid and secondary metabolite metabolism, than PICRUSt. The number of significant pathways predicted by PICRUSt were overestimated in all but carbohydrate metabolism, indicating that CowPI predictions more closely resemble true functionality in secondary rumen metabolism pathways such as amino acid and energy metabolism (Supplementary Tables S1–S3).

Principal component analysis of regularized transformed raw KO counts showed clear separation between time points in the metatranscriptome dataset. Greater separation of 1 and 2 h samples from 4, 6, and 8 h samples follows the previously reported biphasic temporal colonization pattern seen in taxonomic analysis of the samples (Huws et al., 2016). This supports the hypothesis that the metabolic function of the primary forage colonizers differs from that of the secondary colonizers (Huws et al., 2016). Although 16S rRNA data in this study originates from RNA (and therefore represents the metabolically active attached rumen community), and that the KOs only identified in all three datasets were used in the comparison, such stark separation is not seen in either the PICRUSt or CowPI predicted metabolic function. Counts for KOs are lower in the metatranscriptome dataset highlighting that such data represents a transcriptomic snapshot containing only the genes being transcribed at the point at which the sample was taken. The complete genomic content of the fully sequenced organisms used in the inference contain KOs that may have temporally specific transcription patterns. The inclusion of all these genes in the functional inferences made by both CowPI and PICRUSt is likely to have resulted in the lower sensitivity observed. Any functional differences predicted by these tools related to the biphasic nature of forage colonization are likely to represent the taxonomic differences between the primary and secondary colonizing communities.

While CowPI is an improvement over PICRUSt for the inference of function of the rumen microbiome, more accurately highlighting significant differences in represented metabolic pathways, broad comparisons of the three datasets serve to highlight the shortcomings of any results based on inferred or potential function. Arguably, this point can be carried forward to include the use of full metagenomic analyses. This is because the genes identified in such analyses are not necessarily being transcribed at that point in time or in response to an environmental or dietary factor and may only be differentially abundant due to the abundance of the rumen organism containing the gene within its genome. Metagenomic investigation of microbiomes currently represents a complex and involved process. The software tool PICRUSt offers researchers a way of utilizing the simpler, and often more financially viable, 16S rDNA amplicon library approach of community profiling to gain further insight into the predicted functional potential of the studied microbiome and allows researchers to form functional based hypotheses on which future studies can be based. Provided that the shortcomings of such predictive tools, and the inferred results they produce, are recognized by the researchers using them and by the wider scientific community they remain an invaluable tool. These predictive tools maximize the findings from smaller experiments and pilot projects and aid researchers who may not have access to the more advanced computational resources required for full metagenomic analyses. Despite the limitations, as more data is made publicly available and as more knowledge is gained about taxa in the underlying databases, the accuracy of PICRUSt and CowPI functional predictions will only continue to improve.

## Conclusion

In this study, we present the CowPI galaxy workflow, consisting of an OTU table conversion tool and a set of “rumen-specific” pre-calculated files suitable for use in the standard metagenome prediction workflow in PICRUSt. The underlying taxonomic data originates from 16S rRNA gene amplicon sequencing of rumen samples from around the globe and the fully sequenced genomes that have been provided to the Hungate1000 project as cultures isolated from the rumen environment. The CowPI dataset better captures the higher degree of genetic variation and larger pangenomes of rumen organisms. Additionally, we show that in an experimental context, with an underlying question relating to the function of the rumen microbiome, the tool more accurately predicts potential functionality of rumen communities with less genomic “noise” in the form of spuriously predicted metabolic pathways and functions. The tool and underlying data is accessible at http://www.cowpi.org and is provided freely to the wider scientific community for use in the study of the rumen microbiome.

## Author Contributions

The idea for the CowPI tool was conceived by CC and SH. Annotation of bacterial genomes was carried out by TW, CC, and MH. Precalculated files and table conversion workflow was developed by TW and CC. The experiment and sampling used to generate the metatranscriptomic data used in the comparitive analysis was conceived and carried out by SH, JE, and AK-S. Annotation of metagenomic data was performed by KS-T and CC. Processing assembly and annotation of metatranscriptomic data performed by FR and CC. Validation and comparative analyses were carried out by TW. The CowPI galaxy tool is maintained by MF and CC. Paper concept and writing by TW, SH, and CC.

## Conflict of Interest Statement

The authors declare that the research was conducted in the absence of any commercial or financial relationships that could be construed as a potential conflict of interest.

## Footnotes

1 https://www.uniprot.org/uploadlists/

2 www.jgi.doe.gov
